# The GA 20-Oxidase Encoding Gene *MSD1* Controls the Main Stem Elongation in *Medicago truncatula*

**DOI:** 10.3389/fpls.2021.709625

**Published:** 2021-08-04

**Authors:** Wanying Li, Qingxia Ma, Pengcheng Yin, Jiangqi Wen, Yanxi Pei, Lifang Niu, Hao Lin

**Affiliations:** ^1^Biotechnology Research Institute, Chinese Academy of Agricultural Sciences, Beijing, China; ^2^College of Life Science, Shanxi University, Taiyuan, China; ^3^Institute for Agricultural Biosciences, Oklahoma State University, Ardmore, OK, United States; ^4^Department of Plant and Soil Sciences, Oklahoma State University, Stillwater, OK, United States

**Keywords:** GA 20-oxidase, *MSD1*, main stem elongation, functional diversification, *Medicago truncatula*

## Abstract

Plant height is an important agronomic trait that is closely related to biomass yield and crop production. Despite legumes comprise one of the largest monophyletic families that are second only to grasses in terms of economic and nutritional values, due to an ancient genome duplication event, most legume plants have complex genomes, thus the molecular mechanisms that determine plant height are less known in legumes. Here, we report the identification and characterization of *MAIN STEM DWARF1* (*MSD1*), which is required for the plant height in the model legume *Medicago truncatula*. Loss of function of *MSD1* leads to severely reduced main stem height but normal lateral branch elongation in *M. truncatula.* Histological analysis revealed that the *msd1-1* main stem has shorter internodes with reduced cell size and number compared with the wild type, indicating that *MSD1* affects cell elongation and cell proliferation. *MSD1* encodes a putative GA 20-oxidase that is expressed at significantly higher levels in the main shoot apex than in the lateral shoot apices, suggesting that *MSD1* expression is associated with its effect on the main stem elongation. UPLC-MS/MS analysis showed that GA_9_ and GA_4_, two identified products of the GA 20-oxidase, were severely reduced in *msd1-1*, and the dwarf phenotype of *msd1-1* could be rescued by supplementation with gibberellic acid GA_3_, confirming that *MSD1* functions as a biologically active GA 20-oxidase. Moreover, we found that disruption of either *MtGA20ox7* or *MtGA20ox8*, homologs of *MSD1*, has little effects on the elongation of the main stem, while the *msd1-1 mtga20ox7-1 mtga20ox8* triple mutants exhibits a severe short main shoot and lateral branches, as well as reduced leaf size, suggesting that *MSD1* and its homologs *MtGA20ox7* and *MtGA20ox8*, redundantly regulate *M. truncatula* shoot elongation and leaf development. Taken together, our findings demonstrate the molecular mechanism of *MSD1*-mediated regulation of main stem elongation in *M. truncatula* and provide insights into understanding the functional diversity of GA 20-oxidases in optimizing plant architecture in legumes.

## Introduction

Plant height, mainly confined by stem elongation, is not only a decisive factor that affects plant architecture but also an important agronomic trait that contributes to crop yield (Wang and Li, 2008). Gibberellins (GAs) are a large family of tetracyclic diterpenoid plant hormones that play important roles in multiple plant growth and developmental progresses, including promoting seed germination, stem elongation, flowering, pollen development, as well as fruit growth and firmness (Hedden and Sponsel, 2015; Li et al., 2020). The stem elongation function of GA contributed to the Green Revolution in which mutations in GA biosynthesis or signaling are the basis for semidwarf rice and wheat, respectively (Peng et al., 1999; Sasaki et al., 2002).

The biosynthesis of GAs is a multi-step process divided into three stages (Yamaguchi, 2008; Hedden and Thomas, 2012). In the first stage, biosynthesis of *ent*-kaurene is restricted to plastids and catalyzed successively by *ent*-copalyl diphosphate synthase (CPS) and *ent*-kaurene synthase (KS). In the second stage, *ent*-kaurene is converted to GA_12_ by *ent*-kaurene oxidase (KO) and *ent*-kaurenoic acid oxidase (KAO), both of which are cytochrome P450 enzymes (Magome et al., 2013; Nomura et al., 2013; Regnault et al., 2014). In the final stage, GA_12_ is a substrate for cytoplasm-located gibberellin 20-oxidase (GA20ox) multi-family enzymes and follows a non-13-hydroxylation pathway leading to GA_9_ via GA_15_ and GA_24_, and then GA_9_ is converted to bioactive GA_4_ and GA_7_ by GA 3-hydroxylase (GA3ox) (Lange et al., 1994; Chiang et al., 1995; Xu et al., 1995b; Israelsson et al., 2004).

Impaired GA biosynthesis caused by defects in early-step genes, *CPS*, *KS*, and *KO*, leads to typical GA-deficient mutant phenotypes, including severe dwarfism with greatly impaired fertility (Sun and Kamiya, 1994; Helliwell et al., 1998; Ueguchi-Tanaka et al., 2007; Yamaguchi, 2008; Chen et al., 2014; Regnault et al., 2014; Guo et al., 2020). By contrast, mutation of late-step genes of GA biosynthesis leads to semi-dwarf phenotypes because of the functional redundancy of homologous genes, such as genes coding for GA3ox in Arabidopsis, rice (*Oryza sativa*), *Medicago truncatula* and alfalfa (*Medicago sativa*) (Itoh et al., 2001; Dalmadi et al., 2008; Hu et al., 2008; Zhang et al., 2020). Notably, the semi-dwarf “green revolution” phenotype in rice resulted from a mutation in the *sd1* gene, which encodes a GA 20-oxidase (*OsGA20ox2*) (Sasaki et al., 2002; Spielmeyer et al., 2002). Overexpression of *OsGA20ox1* causes a tall and GA-overproduction phenotype; RNAi-mediated suppression of *OsGA20ox1* results in phenotypes that are similar to those of *sd1*, indicating that these two genes probably have complementary function in GA synthesis (Oikawa et al., 2004). In *Arabidopsis*, *GA20ox1* and *GA20ox2* act partially redundantly to promote plant growth (Rieu et al., 2008). These studies indicate that genes in the GA20ox family are of great value for optimizing plant architecture in agricultural species.

Legumes are second only to grasses in terms of economic and nutritional values, and are the major sources of plant proteins and oils for humans and animals (Graham and Vance, 2003). Studies on several dwarf mutants in pea (*Pisum sativum*), soybean (*Glycine max*), and the model legume *M. truncatula* have suggested that the GA pathway plays a conserved role in controlling plant height in legumes (Yaxley et al., 2001; Li et al., 2018; Guo et al., 2020; Zhang et al., 2020). Notably, despite the *M. truncatula* GA20ox family genes exhibited functional redundancy (Ma et al., 2019), overexpression of *GA20ox* can increase *M. truncatula* plant height and biomass (Wang et al., 2020), suggesting an application potential of the GA20ox family genes for biomass improvement in legumes. Nevertheless, due to the ancient genome duplication event, most legume plants undergo gene duplication and subsequent functional diversification (Shoemaker et al., 2006), thus the biological functions of the GA20ox family genes in legumes remain largely unclear.

In this study, we reported the identification and characterization of a distinct dwarf mutant, *main stem dwarf1-1* (*msd1-1*), in *M. truncatula*, which is defective in the main stem elongation. *MSD1* encodes a putative GA 20-oxidase, catalyzing the late step of GA biosynthesis. Our results demonstrated that *MSD1* specifically controls the main stem elongation in *M. truncatula*, while *MSD1*’s homologs genes *MtGA20ox7* or *MtGA20ox8* show little effects on the elongation of the main stem. However, the *msd1-1 mtga20ox7-1 mtga20ox8* triple mutant exhibits a severely short main shoot and lateral branches, as well as reduced leaf size, suggesting that *MSD1* and its homologs *MtGA20ox7* and *MtGA20ox8* redundantly regulate *M. truncatula* shoot elongation and leaf development.

## Materials and Methods

### Plant Materials and Growth Conditions

*Medicago truncatula* strain R108 was used as the wild type for all experiments described in this study. *msd1-1* (NF5514), *msd1-2* (NF12848), *msd1-3* (NF10524), *msd1-4* (NF21287), *mtga20ox7-1* (NF1343), *mtga20ox7-2* (NF18196), and *mtga20ox8* (NF19184) were identified from a *Tnt1* retrotransposon-tagged mutant collection of *M. truncatula* R108 (Tadege et al., 2008). The *M. truncatula* seeds were scarified with sandpaper and germinated at 4°C for 1 week, then the germinated seeds were planted and grown in soil mix (soil:vermiculite = 1:1) in the greenhouse under the following conditions: 24°C day/22°C night temperature, 16-h day/8-h night photoperiod, and 60–70% relative humidity.

The *msd1-1 mtga20ox7-1*, *msd1-1 mtga20ox8*, *mtga20ox7-1 mtga20ox8* double mutants, and the *msd1-1 mtga20ox7-1 mtga20ox8* triple mutants were generated through genetic crosses and identified on the basis of PCR genotyping in the F2 or F3 segregating population. The primers used to identify the *Tnt1* insertions are listed in Supplementary Table 1.

### Morphological Analysis

For the measurement of the internode length, 20 individual plants of both wild type and *msd1-1* mutant were grown simultaneously in the same greenhouse, and the seventh internode beneath the shoot apex of each plant (8-week-old plants) was measured to calculate the average length. Six internodes of each genotype were randomly selected from the above 20 samples, fresh *M. truncatula* internodes from wild type and mutant plants were fixed in 3.5% (v/v) glutaraldehyde in 25 mM phosphate buffer (pH 7) for 48 h, followed by 1% (w/v) osmium tetroxide in 25 mM phosphate buffer (pH 7) for 2 h, and dehydrated in a graded ethanol series (50, 70, 90, 95, 100%), critical-point dried in liquid CO_2_, mounted on aluminum stubs and sputter coated with gold. The internodes were observed using scanning electron microscope by SU8010 (Hitachi, Japan).

In order to measure the length of internode epidermal cells, 15 cells were randomly selected from the SEM images for both wild type and *msd1-1*, and the lengths were measured by using Image J. The cell number was calculated by the ratio of the average internode length (calculated from a total of 20 internodes) to the average cell length (evaluated from 15 cells). Projected areas of leaves were measured by scanning to generate digital images, followed by analysis using the Image J software.

### Phylogenetic Analysis and Sequences Alignment

Multiple sequences alignment was performed using ClustalW.^1^ Bootstrap values of 1000 permutations for the neighbor-joining phylogenetic tree were performed using MEGA 7.0 software.^2^ Accession numbers used in this study are listed in Supplementary Table 2.

### Quantification of Endogenous GAs

Wild type and *msd1-1* plants were grown in soil for 5 weeks in the greenhouse. The main stems (∼0.3 g) from 10 plants of each genotype were collected and mixed together for GA quantification. The GA contents were determined by the Wuhan Greensword Creation Technology Company, and the analysis was performed as described previously (Chen et al., 2012). Three independent biological replicates and technical replicates were measured for each sample.

### Exogenous GA_3_ Application

Bioactive GA_3_ (SIGMA, Lot: BCBR3974V) was dissolved in ethanol (0.1 M) and diluted with ddH_2_O to 2 mM. The first spray was applied at 7-day-old seedlings after sowing, and the later sprays were performed twice a week for four weeks in total. An equivalent group (*n* = 16) of *msd1-1* mutant plants was treated similarly with a solution without GA_3_ (MOCK) at each time. All *msd1-1* mutants with the treatments (+GA_3_ and MOCK) were grown simultaneously in the same greenhouse. Experiments were repeated twice independently with similar results.

### RNA Extraction and Gene Expression Analysis

Total RNA was extracted from plant tissues using TRIzol Reagent (Invitrogen). cDNA was synthesized by reverse transcription with SuperScript (Invitrogen). Reverse transcription PCR (RT-PCR) was performed using a 2xTaq PCR Master Mix (UPTECH) using *MtActin* as a control. Quantitative RT-PCR was performed as previously described (Wang et al., 2017) with at least three biological and three technical replicates for both the treatment samples and controls. All primers used are listed in Supplementary Table 1.

### Plasmid Construction and Plant Transformation

To generate the constructs used for complementation, an 1134-bp of full-length *MSD1* coding sequence was amplified from *M. truncatula* R108 plant and ligated to the pEarlyGate203 vector to generate *p35S:MSD1* construct. The construct were introduced into *Agrobacterium tumefaciens* by chemical transformation. *A. tumefaciens* strain AGL1 was used for *M. truncatula* transformation (Tadege et al., 2011). All primers used are listed in Supplementary Table 1.

## Results

### Identification of the *M. truncatula msd1* Mutant

**FIGURE 1:**
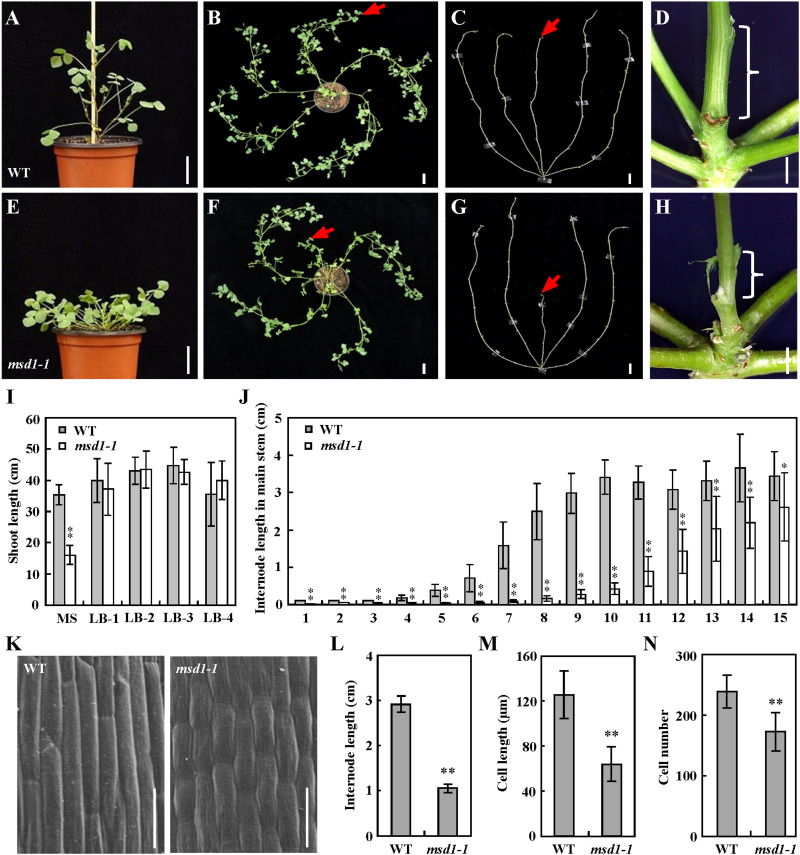
Morphological comparison of the wild type and *msd1-1* plants. **(A–H)** Phenotypic comparison of seedling and stem between wild type (WT) and *msd1-1*. Seedlings of WT **(A,B)** and *msd1-1* **(E,F)** at 5-week-old and 8-week-old, respectively, and 8-week-old bald stems without leaves in WT **(C)** and *msd1-1* **(G)** plants. Bars = 3 cm; panels **(D,H)** are magnifications of shoot base in panels **(C,G)**, respectively. Bars = 2 mm. Red arrows indicate main stem, braces indicate main stem internode. **(I)** Comparison of shoot length between 8-week-old WT and *msd1-1* plants. MS, main stem; LB-1, the first lateral branch; LB-2, the second lateral branch; LB-3, the third lateral branch; LB-4, the fourth lateral branch. Bars represent means ± SD (*n* = 20); asterisks indicate significant differences from the WT (^∗∗^*P* < 0.01, Student’s *t-*test). **(J)** Comparison of internode length between 8-week-old WT and *msd1-1* plants. The x-axis represents corresponding node numbers. Bars represent means ± SD (*n* = 20); asterisks indicate significant differences from the WT (^∗∗^*P* < 0.01, Student’s *t-*test). **(K)** Scanning electron microscope images of the seventh internode of WT and *msd1-1* plants. Bars = 50 μm. **(L)** Comparison of the seventh internode length between 8-week-old WT and *msd1-1* plants. Bars represent means ± SD (*n* = 10); asterisks indicate significant differences from the WT (^∗∗^*P* < 0.01, Student’s *t-*test). **(M,N)** Comparison of cell length **(M)** and cell number **(N)** in the seventh internode between 8-week-old WT and *msd1-1* plants. Bars represent means ± SD (*n* = 15); asterisks indicate significant differences from the WT (^∗∗^*P* < 0.01, Student’s *t-*test).

To investigate the molecular mechanism underlying the regulation of plant height in the model legume *M. truncatula*, we identified a distinct dwarf mutant named *main stem dwarf1-1* (*msd1-1*) from a forward genetic screen of the *Tnt1* retrotransposon-tagged *M. truncatula* mutant population (Yarce et al., 2013). By contrast with the wild type, the *msd1-1* mutant exhibits dwarfed main stem, while side branches were normal (Figures 1A–C,E–G,I). There were no difference in internode number between the *msd1-1* mutant and the wild type, but the length of every internode in the *msd1-1* was significant shorter than that in wild type (Figures 1D,H,J).

To determine the reason of the reduced length in the main stem, we examined the cell numbers and lengths using the seventh main stem internode, which exhibited significant difference, and found that cells in the *msd1*-*1* mutant were ∼50% the length of those in the wild type (Figures 1K,M). The total epidermal cell number was calculated as internode length/epidermal cell length, and this indicated that the number of cells in the *msd1*-*1* mutant was ∼72% that of the wild type (Figures 1L–N). These results revealed that the main stem dwarf phenotype in the *msd1*-*1* mutant resulted from both decreased length and number of internode cells, with the decreases in cell length accounting for the main effect.

### Molecular Cloning of the *MSD1* Gene

**FIGURE 2:**
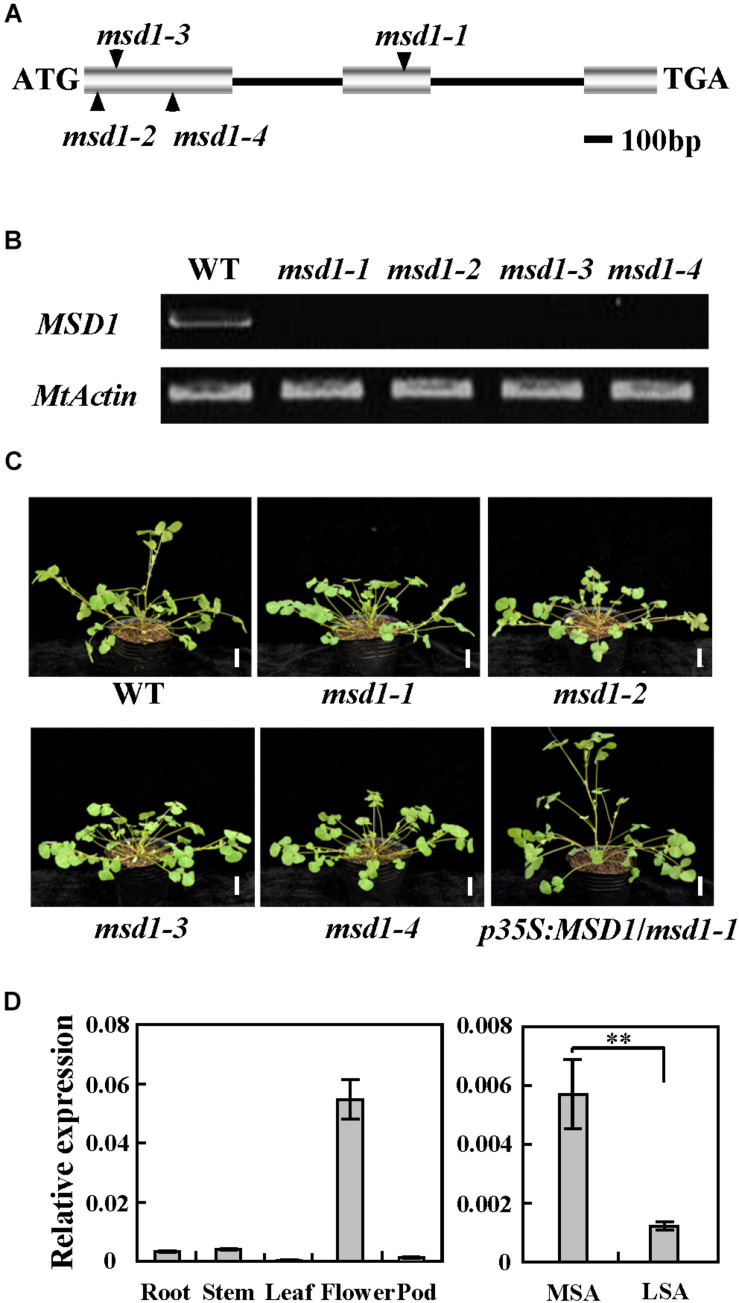
Molecular cloning of the *MSD1* gene. **(A)** Schematic representation of the gene structure of *MSD1* and the *Tnt1* insertion sites in *msd1-1*, *msd1-2, msd1-3*, and *msd1-4*. **(B)** RT-PCR analysis of *MSD1* expression in wild type (WT) and various *msd1* alleles. *MtActin* was used as the loading control. **(C)** Phenotypic analysis of WT, *msd1-1*, *msd1-2, msd1-3, msd1-4*, and *msd1-1* plants complemented with *p35S*:*MSD1*. Bars = 2 cm. **(D)** *MSD1* transcript levels in different tissues, as revealed by quantitative RT-PCR. *MtActin* was used as an internal control. MSA, main shoot apices. LSA, lateral shoot apices. Values are means ± SD of three technical replicates; asterisks indicate significant differences (^∗∗^*P* < 0.01, Student’s *t-*test). Three independent experiments were performed, with similar results.

The *msd1-1* mutant phenotype segregates as a single recessive mutation, in which heterozygous parents produce progeny that segregated 3:1 (35:10) for the wild-type-like and mutant plants. To identify the gene associated with the mutant phenotype, thermal asymmetric interlaced-PCR was performed to recover the flanking sequences of *Tnt1* from *msd1-1* (Tadege et al., 2008). Based on the genotyping results, one *Tnt1* insertion segregating with the mutant phenotype was identified. Further genotyping analyses confirmed that all *msd1-1* mutant plants harbored homozygous insertion for the particular flanking sequence tag (FST). The full length gene sequence corresponding to this FST was recovered and sequence alignment showed that the candidate gene encodes a putative GA 20-oxidase, which is identical to previously reported MtGA20ox1/Medtr1g102070 (Ma et al., 2019). Genomic PCR analysis showed that the *Tnt1* was inserted in the second exon of *MSD1*, resulting in abolished transcription of the full-length *MSD1* (Figures 2A,B). To confirm that the *msd1-1* mutant phenotype is caused by disruption of *MSD1*, we obtained three additional *Tnt1* insertion lines (NF12848, NF10524, and NF21287). Analysis of flanking sequences showed that NF12848, NF10524, and NF21287 contained *Tnt1* insertions at different locations in exon 1 of *MSD1*; we therefore named these lines *msd1-2*, *msd1-3*, and *msd1-4*, respectively (Figure 2A). RT-PCR analysis revealed that the transcripts of *MSD1* were abolished in these four mutants (Figure 2B), and *msd1-2*, *msd1-3*, and *msd1-4* showed similar phenotype as observed in *msd1-1* (Figure 2C). The identity of *MSD1* was further confirmed by genetic complementation. We introduced the 1134-bp of full-length coding sequence of *MSD1* driven by cauliflower mosaic virus (CaMV) *35S* promoter into *msd1-1* plants by *A. tumefaciens*-mediated transformation. The main stem elongation was rescued in the complemented transgenic *msd1-1* plants (Figure 2C). Collectively, these data confirmed that disrupting *MSD1* function leads to the main stem dwarf but normal side branches in the *msd1* mutants.

To explain why the main stem was specifically shortened while the side branches were no significant changes in *msd1-1*, quantitative RT-PCR was conducted in different tissues. The results revealed that *MSD1* was expressed in flowers, axillary buds, stem, root, pods, leaf, and shoot apices (Figure 2D). It is worth mentioning that *MSD1* was expressed significantly higher in main shoot apices than in lateral shoot apices, although the *MSD1* expression level is low in both tissues (Figure 2D), which supports its function in controlling main stem elongation in *M. truncatula*.

### *MSD1* Affects Internode Elongation via Affecting GA Biosynthesis

**FIGURE 3:**
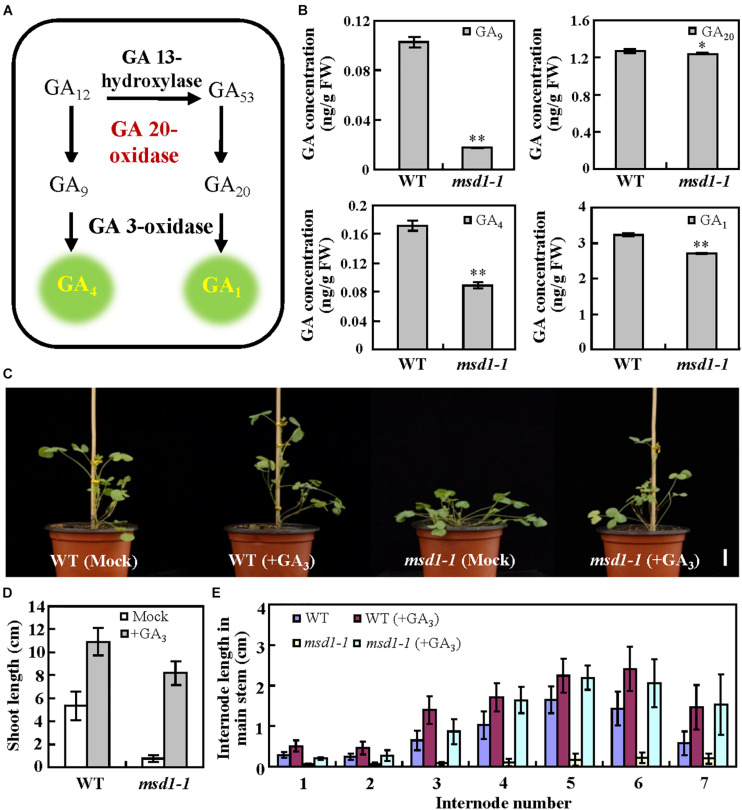
*MSD1* was involved in GA biosynthesis pathway. **(A)** Simplified scheme of the later steps in GA biosynthesis pathway. **(B)** Concentrations of endogenous GA_9_, GA_20_, GA_4_, and GA_1_ in 5-week-old WT and *msd1-1* plants. Bars represent means ± SD (*n* = 3); asterisks indicate significant differences from the WT (^∗^*P* < 0.05, ^∗∗^*P* < 0.01, Student’s *t-*test). **(C)** Phenotypes of wild type (WT) and *msd1-1* mutant treated with 2 mM GA_3_ for 5 weeks in comparison with untreated controls (mock). Bars = 2 cm. (**D**) The main shoot height of plants in the experiment shown in panel **(C)**. Bars represent means ± SD (*n* = 16). **(E)** The internode length in main stem in the experiment shown in panel **(C)**. Bars represent means ± SD (*n* = 16).

Given that GA20ox is a key enzyme of the later steps in the GA biosynthesis pathway (Qin et al., 2013), we speculated that MSD1 might possess the conserved catalyzing function of GA20ox during the synthesis of bioactive GAs in *M*. *truncatula*. It has reported that GA20oxs use C20-GAs as substrates to produce immediate precursors, GA_9_ and GA_20_, then GA_9_ and GA_20_ are respectively converted to bioactive GA_4_ and GA_1_ by GA3ox (Yamaguchi, 2008; Figure 3A). We therefore analyzed the contents of GA_9_, GA_20_, GA_1_ and GA_4_ in *msd1-1*. UPLC-MS/MS analysis showed that the concentration of GA_20_ was slightly reduced in the *msd1-1* mutant while GA_9_ was only 16.8% of that in the wild type (Figure 3B). Meanwhile, GA_1_ and GA_4_ in the *msd1-1* mutant were respectively reduced to 83.8 and 51.6% of wild type (Figure 3B). To investigate whether the GA deficiency is related to the *msd1* mutant phenotype, we treated 5-week-old *msd1-1* mutant plants with 2 mM GA_3_ for a month, and found that the height of the plants was restored compared with the control (Figures 3C–E), indicating exogenous application of GA could restore the dwarf phenotype of the *msd1-1* mutant. Taken together, our results demonstrated that MSD1 regulates the biosynthesis of bioactive GAs, which control plant height in *M*. *truncatula*.

### Phylogenetic Analysis of *M. truncatula* GA 20-Oxidase Family Genes

**FIGURE 4:**
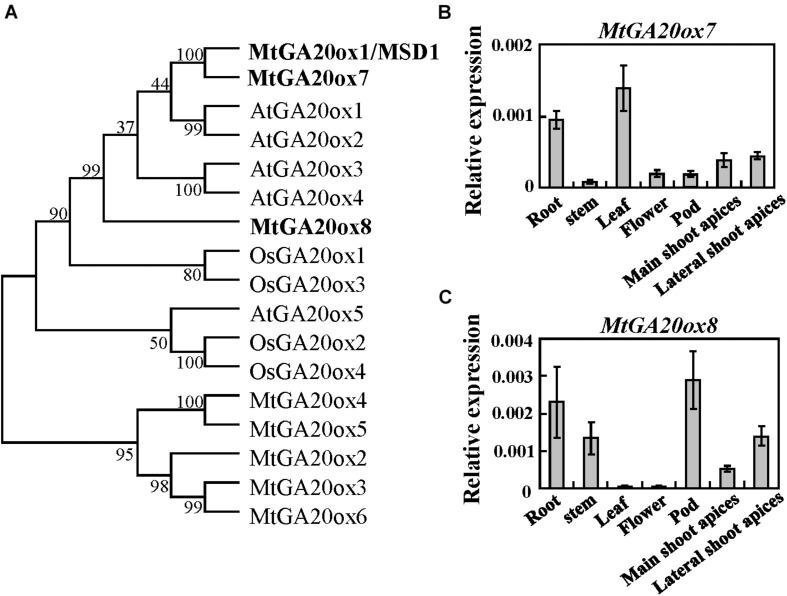
Phylogenetic analysis of MSD1 and its closely related homologs. **(A)** Phylogenetic analysis of MSD1 and its homologs in *M. truncatula* (Mt), *Arabidopsis* (At) and rice (Os). Numbers on branches indicate bootstrap percentages for 1,000 replicates. **(B,C)** *MtGA20ox7-1* and *MtGA20ox8* transcript levels in different tissues, as revealed by quantitative RT-PCR. *MtActin* was used as an internal control. Values are means ± SD of three technical replicates. Three independent experiments were performed, with similar results.

MSD1 belongs to the 2-oxoglutarate-dependent dioxygenase (2-ODDs) family, which contains two conserved domains: DIOX_N and 2OG-FeII_Oxy (Pan et al., 2017; Tenreira and Lange, 2017; Supplementary Figure 1). The protein BLAST analysis revealed that MSD1 has seven homologs in the *M. truncatula* genome. Consistent with the previous report (Ma et al., 2019), phylogenetic analyses suggested MSD1, MtGA20ox7/Medtr6g464620 and MtGA20ox8/Medtr8g033380 were clustered to close groups, with MSD1 and MtGA20ox7 falling within the same clade, which is close to AtGA20ox1-4 (Figure 4A). Quantitative RT-PCR revealed that *MtGA20ox7* and *MtGA20ox8* were both expressed in flowers, axillary buds, stem, root, pods, leaf as well as shoot apices (Figures 4B,C), suggesting that *MtGA20ox7* and *MtGA20ox8* may serve similar function as *MSD1* in the regulation of plant height via affecting GA biosynthesis.

### Genetic Analyses of *MSD1*, *MtGA20ox7*, and *MtGA20ox8* in the Regulation of *M. truncatula* Shoot Development

**FIGURE 5:**
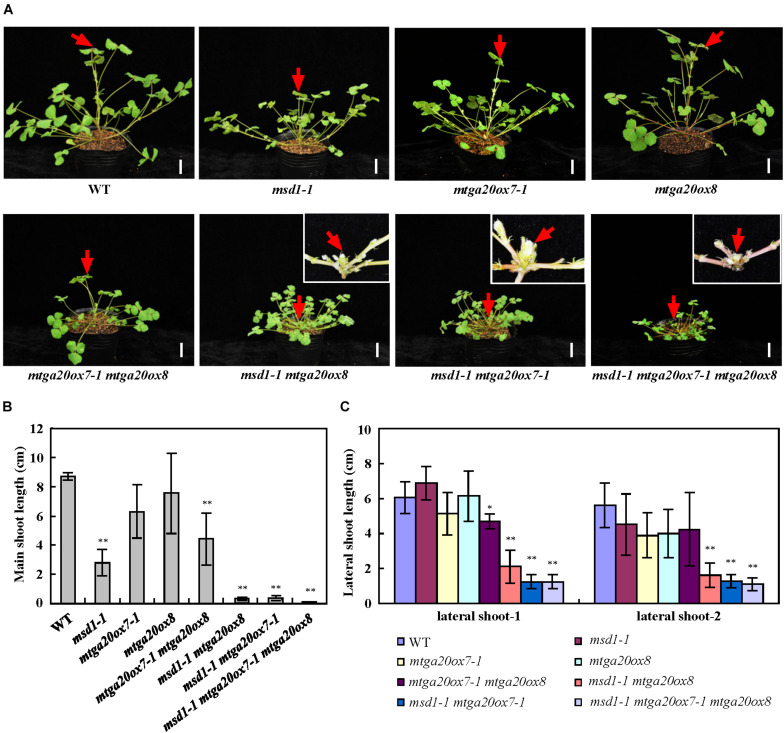
Genetic analyses of *MSD1*, *MtGA20ox7* and *MtGA20ox8* in regulating *M. truncatula* shoot elongation. **(A)** Phenotypic comparison of 4-week-old wild type (WT) and *mtga20* mutants. Red arrows indicate main stem. The insets show close-up views of the main stem. Bars = 2 cm. **(B)** Comparison of the main shoot length in 4-week-old WT and *mtga20* mutants. Bars represent means ± SD (*n* = 16); asterisks indicate significant differences from the WT (^∗∗^*P* < 0.01, Student’s *t-*test). **(C)** Comparison of the lateral shoot length in 4-week-old WT and *mtga20* mutants. Bars represent means ± SD (*n* = 16); asterisks indicate significant differences from the WT (^∗^*P* < 0.05, ^∗∗^*P* < 0.01, Student’s *t-*test).

**FIGURE 6:**
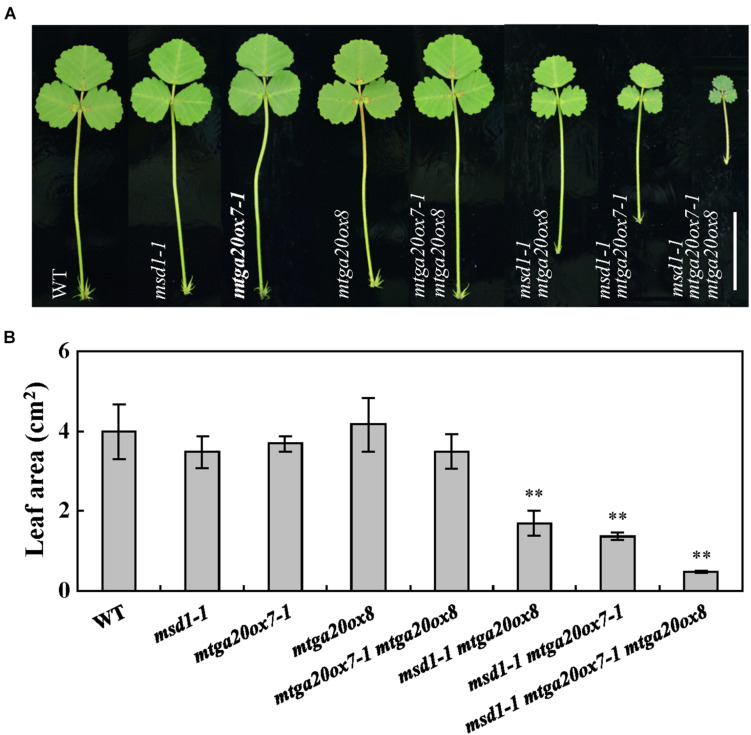
Comparison of leaves in wild type and *mtga20ox* mutants. **(A)** Phenotype of leaves in 4-week-old wild type (WT) and diverse *mtga20ox* mutants. Bars = 2 cm. **(B)** Comparison of leaf size in WT and *mtga20ox* mutants. Bars represent means ± SD (*n* = 16); asterisks indicate significant differences from the WT (^∗∗^*P* < 0.01, Student’s *t-*test).

To investigate the roles of *MtGA20ox7* and *MtGA20ox8* with respect to *MSD1* function in the regulation of shoot elongation, we first identified two mutant lines (NF1343 and NF18196) harboring the *Tnt1* insertion in the *MtGA20ox7* locus (Ma et al., 2019). Analysis of flanking sequences showed that NF1343 and NF18196 contained *Tnt1* insertions in exon 1 and exon 2 of *MtGA20ox7*, respectively (Supplementary Figure 2A). We therefore named these two lines *mtga20ox7-1* and *mtga20ox7-2*. RT-PCR analysis revealed that the transcripts of *MtGA20ox7* were abolished in above two mutant lines (Supplementary Figure 2B). Meanwhile, a loss-of-function mutant line (NF19184, named *mtga20ox8*) harboring the *Tnt1* insertion in *MtGA20ox8* was also identified (Ma et al., 2019; Supplementary Figure 3). In agree to previous findings, no obvious plant height defects were observed in *mtga20ox7-1*, *mtga20ox7-2*, and *mtga20ox8* compared to the wild-type plants (Ma et al., 2019; Figure 5A; Supplementary Figures 2C, 3C). Next, we generated double and triple mutants with combinations of *msd1-1*, *mtga20ox7-1*, and *mtga20ox8* to investigate the genetic relationship among *MSD1*, *MtGA20ox7*, and *MtGA20ox8* in *M. truncatula* shoot elongation. Consistent with the notion that *MSD1* acts as an essential regulator in *M. truncatula* main stem elongation, the *msd1-1* showed the most severe phenotype, with significantly reduced main stem elongation relative to the *mtga20ox7-1* and *mtga20ox8* single mutants, which are comparable to wild type. However, the *mtga20ox7-1 mtga20ox8* double mutants exhibited reduced main stem height, indicating that the contributions of *MtGA20ox7* and *MtGA20ox8* to *M. truncatula* main stem elongation are secondary and redundant (Figures 5A,B). Notably, the *msd1-1 mtga20ox7-1* and *msd1-1 mtga20ox8* double mutants and the *msd1-1 mtga20ox7-1 mtga20ox8* triple mutant exhibited drastically reduced main stem height, showing an additive defect on main stem elongation (Figures 5A,B). In addition, phenotypic observations showed lateral shoot length and leaf size was reduced in both *msd1-1 mtga20ox7-1* and *msd1-1 mtga20ox8* double mutants, but the *msd1-1 mtga20ox7-1 mtga20ox8* triple mutant showed drastically reduced lateral shoot length and leaf size relative to wild type plants (Figures 5C, 6). Collectively, these results indicated that *MSD1* and its homologs *MtGA20ox7* and *MtGA20ox8* coordinately regulate shoot elongation and leaf development in *M. truncatula*.

## Discussion

Plant height is one of the most important agricultural traits that determines biomass production and grain yield (Salas Fernandez et al., 2009). Despite the genes encoding GA20ox have been identified and characterized in several plant species (Rieu et al., 2008; Asano et al., 2011; Plackett et al., 2012; Chen et al., 2019), relatively little progress regarding the biological function of the GA20ox family genes is demonstrated in legumes with complex genomes (Igielski and Kepczynska, 2017). In this study, we reported that the disruption of *MSD1*, a putative GA 20-oxidase, by *Tnt1* retrotransposon insertion resulted in severely reduced main stem elongation, which is associated with reduced content of GA in the model legume *M. truncatula* (Figures 2A,B), suggesting the functional conservation of the GA20ox family genes in the regulation of plant height in legumes. The contents of the bioactive GA_1_ and GA_4_ were both reduced in the *msd1* mutant compared with the wild type (Figure 3B), indicating that MSD1 catalyzed the synthesis of bioactive GAs. Nevertheless, in contrast to GA_20_ and GA_1_, which show a relatively small reduction, the concentrations of GA_9_ and GA_4_ were significantly reduced in the *msd1* mutant, suggesting that MSD1 may have a much greater effect on the biosynthesis of non-13-hydroxylated GAs in *M. truncatula*. Histological analysis showed the main shoot dwarf phenotype of *msd1* is caused by the decrease of the cell elongation and cell division in the main stem (Figures 1K–N). This is consistent with previous studies demonstrating that GAs enhance cell elongation and proliferation (de Lucas et al., 2008; Hedden and Thomas, 2012; Lee et al., 2012; Tong et al., 2014). It has been reported that the cell elongation is regulated by cell wall-loosening protein expansin (EXP) and xyloglucan endo-transglycosylases (XET), which have been shown to be specifically upregulated by GAs in *Arabidopsis* and rice (Xu et al., 1995a; Lee and Kende, 2001, 2002). In addition, GAs can also induce cell elongation via upregulating the transcription levels of cell division-related genes including cell cycle genes *CYCA1;1* and *CDC2Os-3* in deepwater rice (Lee and Kende, 2002). Nevertheless, so far, the underlying mechanism by which GA regulates the expression of these genes remains to be elucidated in legume plants. Thus, the identification of *msd1* mutant may provide a model system to further investigate the regulation mechanism of GA in determining cell proliferation and elongation in *M. truncatula* and other legumes.

It is worth noting that the *msd1* plant showed a severely reduced main stem height but with normal lateral branch elongation (Figures 1A–J), which is rarely observed in *GA20ox* mutants identified in other plant species, suggesting the functional diversity of GA20ox in regulating plant height in legumes. The specific function of *MSD1* in controlling main stem elongation may be explained by its spatial expression profile. Quantitative RT-PCR analysis revealed that *MSD1* is expressed at significantly higher levels in the main shoot apex than in the lateral shoot apices, suggesting that tissue specificity appears to be important for the functional diversification among *GA20ox* gene family. Nevertheless, the finding that differential expression of *MSD1* in main and lateral branches leads to significant different shoot length may provide a cue for further investigating the regulation mechanism of GA in *M. truncatula* shoot development and may be valuable in plant breeding.

The *M. truncatula* genome contains eight GA20ox family members and phylogenetic analyses showed that MSD1, MtGA20ox7 and MtGA20ox8 are close to AtGA20ox1-4, OsGA20ox1 and OsGA20ox3, which are involved in regulating plant height in Arabidopsis and rice (Figure 4A; Igielski and Kepczynska, 2017). Phenotypic analysis in single mutant of *msd1-1, mtga20ox7-1* and *mtga20ox8* suggested that *MSD1* plays an essential role in controlling main shoot height, while *MtGA20ox7* and *MtGA20ox8* showed no obvious influence in regulating main shoot height in *M. truncatula* (Figures 5A,B and Supplementary Figures 2C, 3C). Nevertheless, the double mutants of *msd1-1 mtga20ox7-1* and *msd1-1 mtga20ox8* both exhibit more severe dwarf in main shoot height compared to *msd1-1* (Figures 5A,B). The triple mutant *msd1-1 mtga20ox7-1 mtga20ox8* has the most seriously decreased main shoot height and leaf size (Figures 5A,B, 6), suggesting that *MtGA20ox7* and *MtGA20ox8* are functionally redundant to *MSD1* in the regulation of shoot elongation and leaf development. Therefore, further elucidating the actions of diverse MtGA20ox members and investigating their genetic interactions will enlighten our understanding of biological function of *MtGA20ox*s in controlling *M. truncatula* growth and development.

Taken together, our studies reveal the molecular mechanism of *MSD1*-mediated regulation of main stem elongation and demonstrate the coordination of *MSD1* and its homologs *MtGA20ox7* and *MtGA20ox8* in controlling *M. truncatula* shoot elongation and leaf development, which provide insights into understanding the functional diversity of GA 20-oxidases in optimizing plant architecture in legumes.

## Data Availability Statement

The original contributions presented in the study are included in the article/Supplementary Material, further inquiries can be directed to the corresponding author/s.

## Author Contributions

WL, QM, PY, LN, and HL designed the research and analyzed the data. WL, QM, and PY performed the experiments. JW and YP contributed the analytical tools. WL, LN, and HL wrote the manuscript. All authors contributed to the article and approved the submitted version.

## Conflict of Interest

The authors declare that the research was conducted in the absence of any commercial or financial relationships that could be construed as a potential conflict of interest.

## Publisher’s Note

All claims expressed in this article are solely those of the authors and do not necessarily represent those of their affiliated organizations, or those of the publisher, the editors and the reviewers. Any product that may be evaluated in this article, or claim that may be made by its manufacturer, is not guaranteed or endorsed by the publisher.

## Footnotes

1
http://www.genome.jp/tools/clustalw/

2
http://www.megasoftware.net/

## Supplementary Material

Supplementary Figure 1 Amino acid sequence alignment of MSD1 and its close homologs in Arabidopsis and *M. truncatula*.

Supplementary Figure 2 Identification of the *mtga20ox7* mutant.

Supplementary Figure 3 Identification of the *mtga20ox8* mutant.

Supplementary Table 1 Primers used in this study.

Supplementary Table 2 Accession numbers used in this study.
