# Supplementary material for: The GA 20-Oxidase Encoding Gene MSD1 Controls the Main Stem Elongation in Medicago truncatula
Source: Front Plant Sci. 2021 Aug 4;12:709625. doi: 10.3389/fpls.2021.709625 (PMC8371406; doi:10.3389/fpls.2021.709625)
Supplement: Supplementary Figure 1 — Amino acid sequence alignment of MSD1 and its close homologs in Arabidopsis and M. truncatula. [file Data_Sheet_1.PDF]

```
MSD1        1   MAIECITSMPQQLLNQET----KEQEKPLVFDASVLKHQVN-LPTQFIWPDEEQACLNVPELDVPFIDLG 65
AtGA20ox1   1   MAVSFVTTSPEE-EDKPKLGLGNIQT-PLIFNPSMLNLQAN-IPNQFIWPDDEKPSINVLELDVPLIDLQ 67
AtGA20ox2   1   MAILCTTTSPAEKEHEPKQDLEKDQTSPLIFNPSLLNLQSQ-IPNQFIWPDEEKPSIDIPELNVPFIDLS 69
AtGA20ox3   1   MATECIATVPQIFSENKT-----KEDSS-IFDAKLLNQHSHHIPQQFVWPDHEKPSTDVQPLQVPLIDLA 64
AtGA20ox4   1   --MECIIKLPQRFNKNKS-----KKNPLRIFDSTVLNHQPDHIPQEFVWPDHEKPSKNVPILQVPVIDLA 63

MSD1        66  GFLSGDPVAAMEASKVVGEACQKHGFFLVVNHGIDEKLISDAHVFMDDFFELPLSQKQRAQRKTGEHCGY 135
AtGA20ox1   68  NLLS-DPSSTLDASRLISEACKKHGFFLVVNHGISEELISDAHEYTSRFFDMPLSEKQRVLRKSGESVGY 136
AtGA20ox2   69  -----SQDSTLEAPRVIAEACTKHGFFLVVNHGVSESLIADAHRLMESFFDMPLAGKQKAQRKPGESCGY 134
AtGA20ox3   65  GFLSGDSCLASEATRLVSKAATKHGFFLITNHGVDESLLSRAYLHMDSFFKAPACEKQKAQRKWGESSGY 134
AtGA20ox4   64  GFLSNDPLLVSEAERLVSEAAKKHGFFLVTNHGVDERLLSTAHKLMDTFFKSPNYEKLKAQRKVGETTGY 133

MSD1        136 ASSFTGRFSSKLPWKETLSFQFSADEKS---PNLVRDYLCNTMGNEFEKFGDVYQDYCKAMSNLSLGIME 202
AtGA20ox1   137 ASSFTGRFSTKLPWKETLSFRFCDDMSR---SKSVQDYFCDALGHGFQPFGKVYQEYCEAMSSLSLKIME 203
AtGA20ox2   135 ASSFTGRFSTKLPWKETLSFQFSNDNSG---SRTVQDYFSDTLGQEFEQFGKVYQDYCEAMSSLSLKIME 201
AtGA20ox3   135 ASSFVGRFSSKLPWKETLSFKFSPEEK--IHSQTVKDFVSKKMGDGYEDFGKVYQEYAEAMNTLSLKIME 202
AtGA20ox4   134 ASSFVGRFKENLPWKETLSFSFSPTEKSENYSQTVKNYISKTMGDGYKDFGSVYQEYAETMSNLSLKIME 203

MSD1        203 ILGMSLGVGKAHFREFFEENSSIMRLNYYPTCQKPELTLGTGPHCDPTSLTILHQDQVGGLQVYVDDQWH 272
AtGA20ox1   204 LLGLSLGVKRDYFREFFEENDSIMRLNYYPPCIKPDLTLGTGPHCDPTSLTILHQDHVNGLQVFVENQWR 273
AtGA20ox2   202 LLGLSLGVNRDYFRGFFEENDSIMRLNHYPPCQTPDLTLGTGPHCDPSSLTILHQDHVNGLQVFVDNQWQ 271
AtGA20ox3   203 LLGMSLGVERRYFKEFFEDSDSIFRLNYYPQCKQPELALGTGPHCDPTSLTILHQDQVGGLQVFVDNKWQ 272
AtGA20ox4   204 LLGMSLGIKREHFREFFEDNESIFRLNYYPKCKQPDLVLGTGPHCDPTSLTILQQDQVSGLQVFVDNQWQ 273

MSD1        273 SISPHFNAFVVNIGDTFMALSNGRYKSCLHRAVVNSEKTRKSLAFFLCPLSDKVVTPPCELVDN--YNPR 340
AtGA20ox1   274 SIRPNPKAFVVNIGDTFMALSNDRYKSCLHRAVVNSESERKSLAFFLCPKKDRVVTPPRELLDS--ITSR 341
AtGA20ox2   272 SIRPNPKAFVVNIGDTFMALSNGIFKSCLHRAVVNRESARKSMAFFLCPKKDKVVKPPSDILEK--MKTR 339
AtGA20ox3   273 SIPPNPHAFVVNIGDTFMALTNGRYKSCLHRAVVNSERERKTFAFFLCPKGEKVVKPPEELVNGVKSGER 342
AtGA20ox4   274 SIPPIPQALVVNIGDTLMALTNGIYKSCLHRAVVNGETTRKTLAFFLCPKVDKVVKPPSELE-----GER 338

MSD1        341 IYPDFTWSMLLEFTQKHYRADIKTLEAFAKWVQCKST-- 377
AtGA20ox1   342 RYPDFTWSMFLEFTQKHYRADMNTLQAFSDWLTK---PI 377
AtGA20ox2   340 KYPDFTWSMFLEFTQKHYRADVNTLDSFSNWVITNNNPI 378
AtGA20ox3   343 KYPDFTWSMFLEFTQKHYRADMNTLDEFSIWLKNRRSF- 380
AtGA20ox4   339 AYPDFTWSMFLEFTMKHYRADMNTLEEFTNWLKNKGSF- 376
```

FIGURE S1. Amino acid sequence alignment of MSD1 and its close homologs in Arabidopsis.

Alignment of MSD1 with the four AtGA20ox was performed using ClustalW. The red line represents DIOX_N domain. The black line represents 2OG-FeII_Oxy domain.

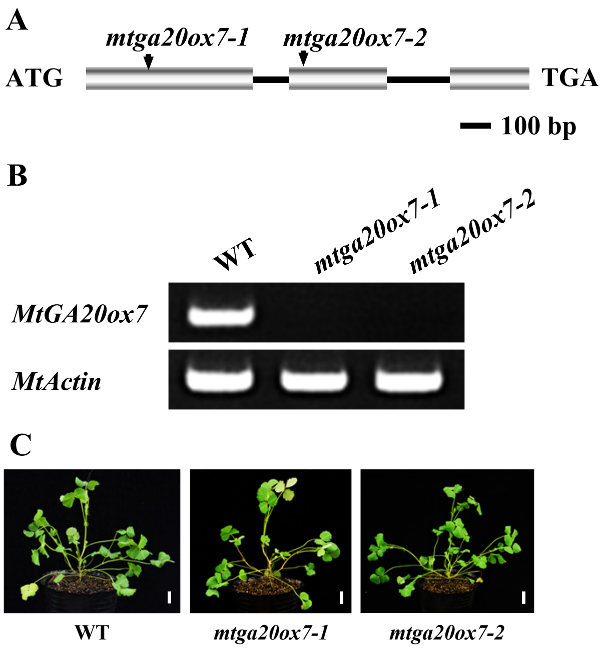

FIGURE S2. Identification of the *mtga20ox7* mutant.

(**A**) Schematic representation of the gene structure of *MtGA20ox7* and the *Tnt1* insertion sites in *mtga20ox7-1* and *mtga20ox7-2*. (**B**) RT-PCR analysis of *MtGA20ox7* expression in wild type (WT) and various *mtga20ox7* alleles. *MtActin* was used as the loading control. (**C**) Phenotypic analysis of 4-week-old WT, *mtga20ox7-1* and *mtga20ox7-2*. Bars = 2 cm.

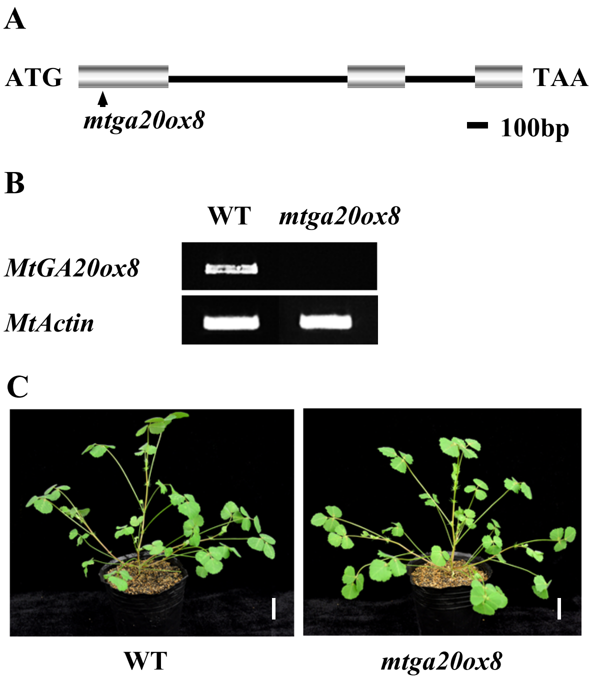

FIGURE S3. Identification of the *mtga20ox8* mutant.

(**A**) Schematic representation of the gene structure of *MtGA20ox8* and the *Tnt1* insertion sites in *mtga20ox8*. (**B**) RT-PCR analysis of *MtGA20ox8* expression in wild type (WT) and *mtga20ox8* mutant. *MtActin* was used as the loading control. (**C**) Phenotypic analysis of 4-week-old WT and *mtga20ox8*. Bars = 2 cm.

Table S1. Primers used in this study.

Primers	Sequences 5'-3'	Application
NF5514-F	CTTGGAGTTGGTAAAGCTCA	For genotyping of NF5514
NF5514-R	AAATGAACAGAGCCCTAACC	
NF12848-F	CTTGGCGTTTCTTTCATCTC	For genotyping of NF12848
NF12848-R	TGTTGTGTTAGGATGCAGTT	
NF10524-F	CTTGGCGTTTCTTTCATCTC	For genotyping of NF10524
NF10524-R	TGTTGTGTTAGGATGCAGTT	
NF21287-F	CTTGGCGTTTCTTTCATCTC	For genotyping of NF21287
NF21287-R	TGTTGTGTTAGGATGCAGTT	
NF1343-F	AGGCACACCATTAACCTTCC	For genotyping of NF1343
NF1343-R	TTGGTCTTGGTGTAGAATGG	
NF18196-F	AGGCACACCATTAACCTTCC	For genotyping of NF18196
NF18196-R	TTGGTCTTGGTGTAGAATGG	
NF19184-F	GGCCATTAATGCATGTTTGA	For genotyping of NF19184
NF19184-R	TTCTCCAACCTTCCTTTGAG	
LTR6	GCTACCAACCAAACCAAGTCAA	Primers in Tnt1for genotyping of different mutant
LTR31	CTCCTCTCGGGGTCGTGGTT	
RT-MSD1-F	ATGGCAATAGAATGCATAAC	For cloning of the *MSD1* CDS and RT-PCR
RT-MSD1-R	TCATGTGCTTTTACATTGAA	
RT-MtGA20ox7-F	ATGGCTATAGAGTGCATAAC	For cloning of the *MtGA20ox7* CDS and RT-PCR
RT-MtGA20ox7-R	TCAGCTACTTTTTTGTTGGA	
RT-MtGA20ox8-F	ATGCATGTCCTTAACCCTTC	For cloning of the *MtGA20ox8* CDS and RT-PCR
RT-MtGA20ox8-R	TTAGTTGAGTTGTTTTTCTC	
RT-MtActin-F	TCTTACTCTCAAGTACCCCATTGAGC	For RT-PCR analysis of *MtActin*
RT-MtActin-R	GTGGGAGTGCATAACCCTCATAGATT	
qMtActin-F	TCAATGTGCCTGCCATGTATGT	For qRT-PCR analysis of *MtActin*
qMtActin-R	ACTCACACCGTCACCAGAATCC	
qPCR-MSD1-F	ACCTTGTCAGAGACTATTTGTG	For qRT-PCR analysis of *MSD1*
qPCR-MSD1-R	AGTAGGATAGTAATTGAGCCTCA	
qPCR-MtGA20ox7-F	AATTTCCTTTCTGGTGACCCTT	For qRT-PCR analysis of *MtGA20ox7*
qPCR-MtGA20ox7-R	AAAGGTAGTCTTTAACAATATTTGT	
qPCR-MtGA20ox8-F	TCCACCAAGATCAAGTTGAGGG	For qRT-PCR analysis of *MtGA20ox8*
qPCR-MtGA20ox8-R	GCGCCATAAATGTGTCACCA	

Table S2. Accession numbers used in this study.

**Gene name and species**	**Accession numbers**
MtGA20ox1/MSD1 (*Medicago truncatula*)	Medtr1g102070
MtGA20ox2 (*Medicago truncatula*)	Medtr3g096500
MtGA20ox3 (*Medicago truncatula*)	Medtr8g093930
MtGA20ox4 (*Medicago truncatula*)	Medtr1g081840
MtGA20ox5 (*Medicago truncatula*)	Medtr3g088745
MtGA20ox6 (*Medicago truncatula*)	Medtr8g093980
MtGA20ox7 (*Medicago truncatula*)	Medtr6g464620
MtGA20ox8 (*Medicago truncatula*)	Medtr8g033380
AtGA20ox1 (*Arabidopsis thaliana*)	At4g25420
AtGA20ox2 (*Arabidopsis thaliana*)	At5g51810
AtGA20ox3 (*Arabidopsis thaliana*)	At5g07200
AtGA20ox4 (*Arabidopsis thaliana*)	At1g60980
AtGA20ox5 (*Arabidopsis thaliana*)	At1g44090
OsGA20ox1 (*Oryza sativa*)	Os03g0856700
OsGA20ox2 (*Oryza sativa*)	Os01g0883800
OsGA20ox3 (*Oryza sativa*)	Os07g0169700
OsGA20ox4 (*Oryza sativa*)	Os05g0421900
